# Supplementary material for: The association between temperature and alcohol- and substance-related disorder hospital visits in New York State
Source: Commun Med (Lond). 2023 Sep 26;3:118. doi: 10.1038/s43856-023-00346-1 (PMC10522658; doi:10.1038/s43856-023-00346-1)
Supplement: Supplementary file 1 — Supplementary Information [file 43856_2023_346_MOESM1_ESM.pdf]

Supplementary Information

The association between rising temperature and alcohol- and substance-related disorder hospital visits in New York State

Robbie M Parks, Sebastian T Rowland, Vivian Do, Amelia K Boehme, Francesca Dominici, Carl L Hart, Marianthi-Anna Kioumourtzoglou

Supplementary Figure 1. Monthly cannabis, cocaine, opioid, and sedative hospital visits, by cause, sex, and location in New York State for 1995-2014.

Supplementary Figure 2. Exposure-response curves of cumulative percentage change in hospital visit rates relative to minimal temperature (-30.1°C (-22.18°F)) for cannabis, cocaine, opioid, and sedative hospital visits were each of the lag days (0 to 6 days before) at the quoted temperature before hospital visit. Black lines show the point estimates and orange ribbons represent 95% confidence intervals.

Supplementary Figure 3. Percentage change in hospital visit rates by selected percentiles of temperature relative to minimal temperature (-30.1°C (-22.18°F)) for cannabis, cocaine, opioid, and sedative hospital visits by location in New York State, were each of the lag days (0 to 6 days before) at the quoted temperature percentile before hospital visit. Points show the point estimates and whiskers represent 95% confidence intervals.

Supplementary Figure 4. Percentage change in hospital visit rates by selected percentiles of temperature relative to minimal temperature (-30.1°C (-22.18°F)) for alcohol- and substance-related disorder hospital visits by age group, were each of the lag days (0 to 6 days before) at the quoted temperature percentile before hospital visit. Points show the point estimates and whiskers represent 95% confidence intervals.

Supplementary Figure 5. Percentage change in hospital visit rates by selected percentiles of temperature relative to minimal temperature (-30.1°C (-22.18°F)) for alcohol- and substance-related disorder hospital visits by sex, were each of the lag days (0 to 6 days before) at the quoted temperature percentile before hospital visit. Points show the point estimates and whiskers represent 95% confidence intervals.

Supplementary Figure 6. Percentage change in hospital visit rates by selected percentiles of temperature relative to minimal temperature (-30.1°C (-22.18°F)) for alcohol- and substance-related disorder hospital visits by Social Vulnerability Index (SVI) tertile, were each of the lag days (0 to 6 days before) at the quoted temperature percentile before hospital visit. Points show the point estimates and whiskers represent 95% confidence intervals.

Supplementary Figure 7. Percentage change in hospital visit rates by selected percentiles of temperature relative to minimal temperature (-30.1°C (-22.18°F)) for cannabis, cocaine, opioid, and sedative hospital visits by age group, were each of the lag days (0 to 6 days before) at the quoted temperature percentile before hospital visit. Points show the point estimates and whiskers represent 95% confidence intervals.

Supplementary Figure 8. Percentage change in hospital visit rates by selected percentiles of temperature relative to minimal temperature (-30.1°C (-22.18°F)) for cannabis, cocaine, opioid, and sedative hospital visits by sex, were each of the lag days (0 to 6 days before) at the quoted temperature percentile before hospital visit. Points show the point estimates and whiskers represent 95% confidence intervals.

Supplementary Figure 9. Percentage change in hospital visit rates by selected percentiles of temperature relative to minimal temperature (-30.1°C (-22.18°F)) for cannabis, cocaine, opioid, and sedative hospital visits by Social Vulnerability Index (SVI) tertile, were each of the lag days (0 to 6 days before) at the quoted temperature percentile before hospital visit. Points show the point estimates and whiskers represent 95% confidence intervals.

Supplementary Figure 10. Exposure-response curve of cumulative percentage change in hospital visit rates relative to minimal temperature (-30.1°C (-22.18°F)) for alcohol- and substance-related disorder hospital visits, were each of the lag days (0 to 1 day before) at the quoted temperature before hospital visit. Black lines show the point estimates and orange ribbons represent 95% confidence intervals.

Supplementary Figure 11. Exposure-response curve of cumulative percentage change in hospital visit rates relative to minimal temperature (-30.1°C (-22.18°F)) for cannabis, cocaine, opioid, and sedative hospital visits, were each of the lag days (0 to 1 day before) at the quoted temperature before hospital visit. Black lines show the point estimates and orange ribbons represent 95% confidence intervals.

Supplementary Figure 12. Comparison of percentage change in hospital visit rates relative to minimal temperature (-30.1°C (-22.18°F)) by selected percentiles (1%, 10%, 25%, 50%, 75%, 90%, 99%) when including or not including relative humidity term ($\sum_{l=0}^{6} s(RH, df)_{lci}$), for a cumulative lag of up to six days before hospital visit. The values from the main model are on the x-axis with values from alternative model with no relative humidity terms on the y-axis. Dots show the point estimates and whiskers represent 95% credible intervals.

Supplementary Figure 13. Map of average temperature by ZIP Code in New York State for 1995-2014.

Supplementary Figure 14. Map of average relative humidity by ZIP Code in New York State for 1995-2014.

Supplementary Figure 15. 2014 Social Vulnerability Index (SVI) tertiles by ZIP Code in New York State. The first tertile (blue) represents lowest social vulnerability and the third tertile (red) represents highest social vulnerability.

Supplementary Table 1. Causes of hospital visit used in the analysis with ICD-9-CM codes.

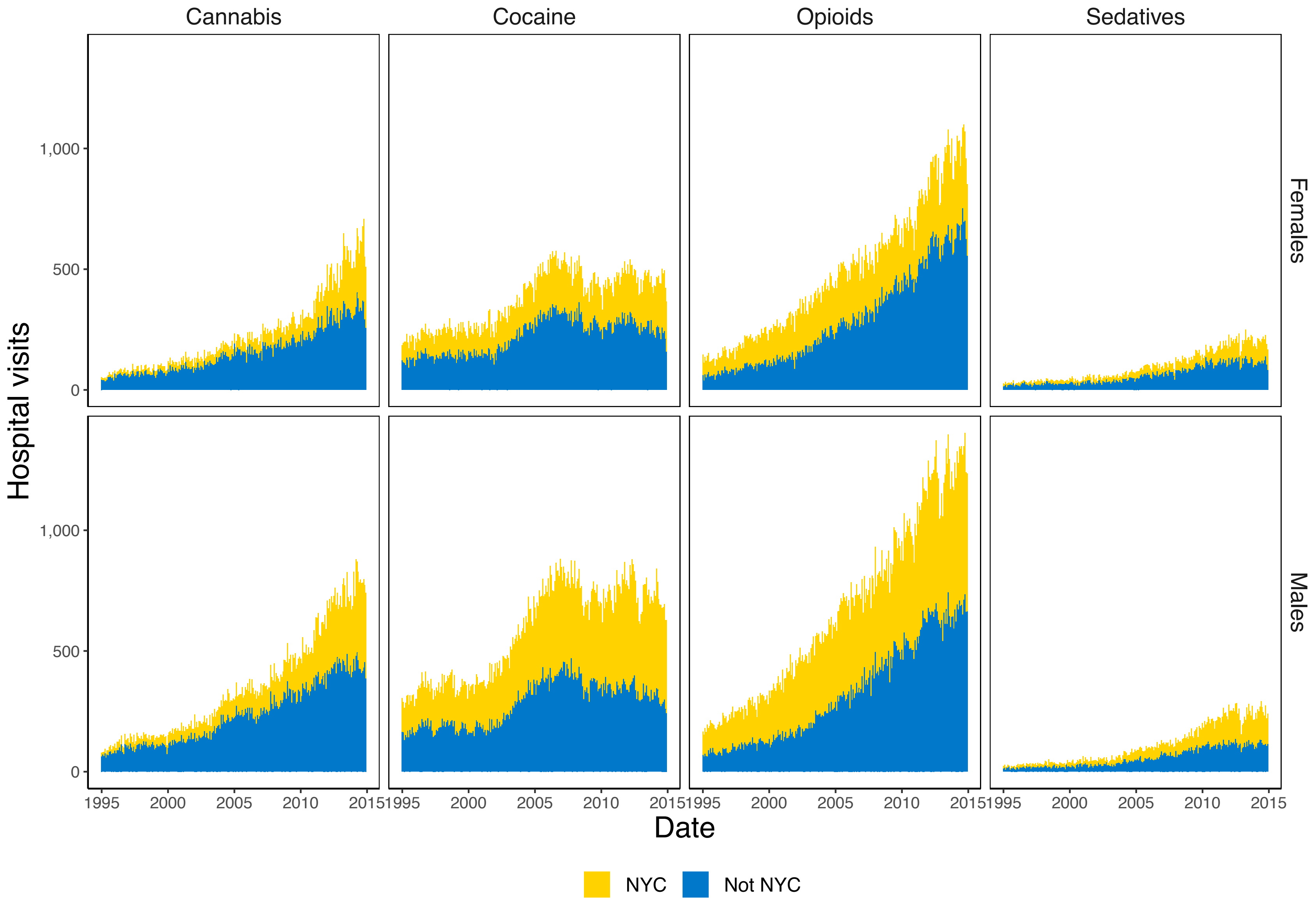

Supplementary Figure 1. Monthly cannabis, cocaine, opioid, and sedative hospital visits, by cause, sex, and location in New York State for 1995-2014.

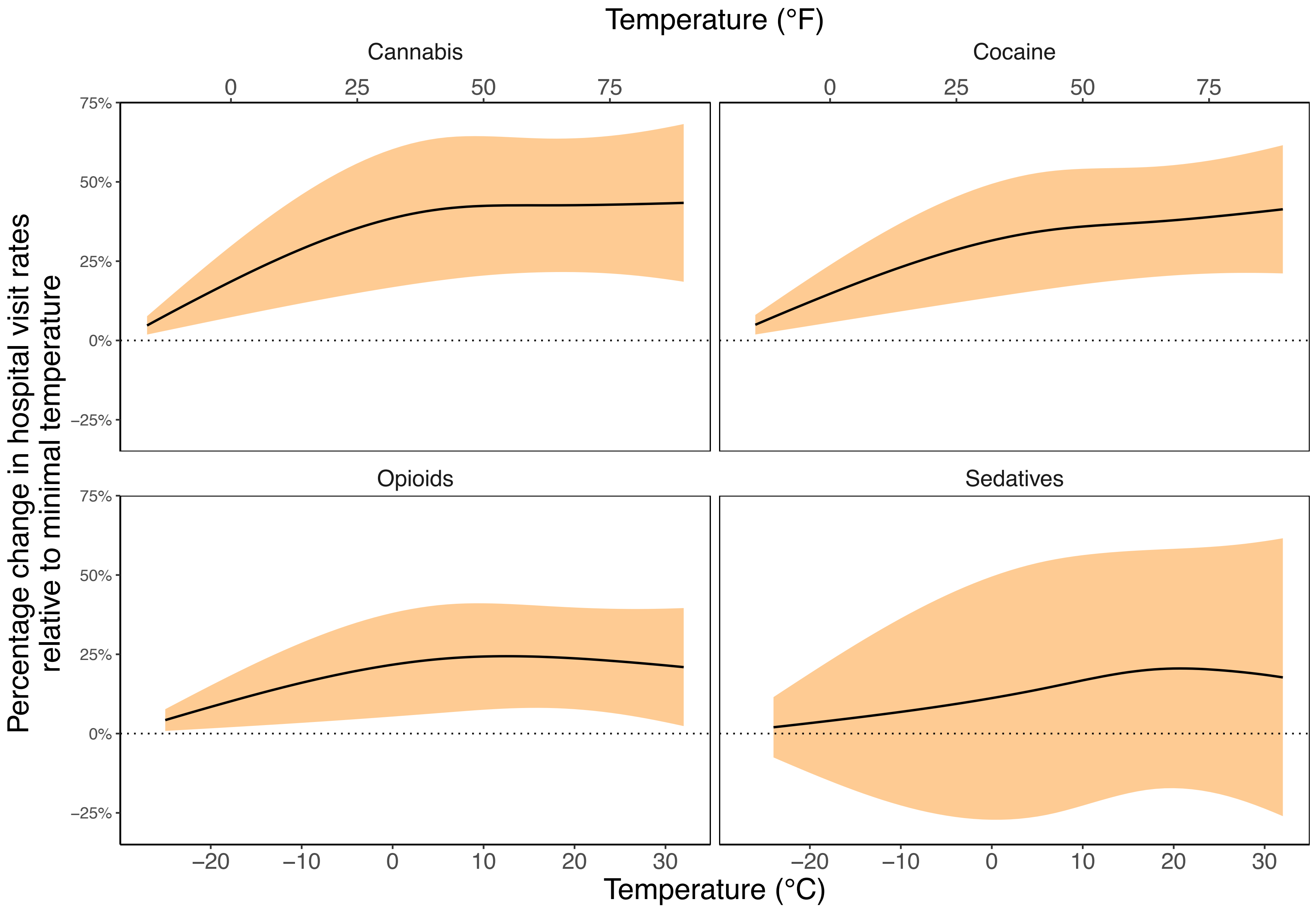

Supplementary Figure 2. Exposure-response curves of cumulative percentage change in hospital visit rates relative to minimal temperature (-30.1°C (-22.18°F)) for cannabis, cocaine, opioid, and sedative hospital visits were each of the lag days (0 to 6 days before) at the quoted temperature before hospital visit. Black lines show the point estimates and orange ribbons represent 95% confidence intervals.

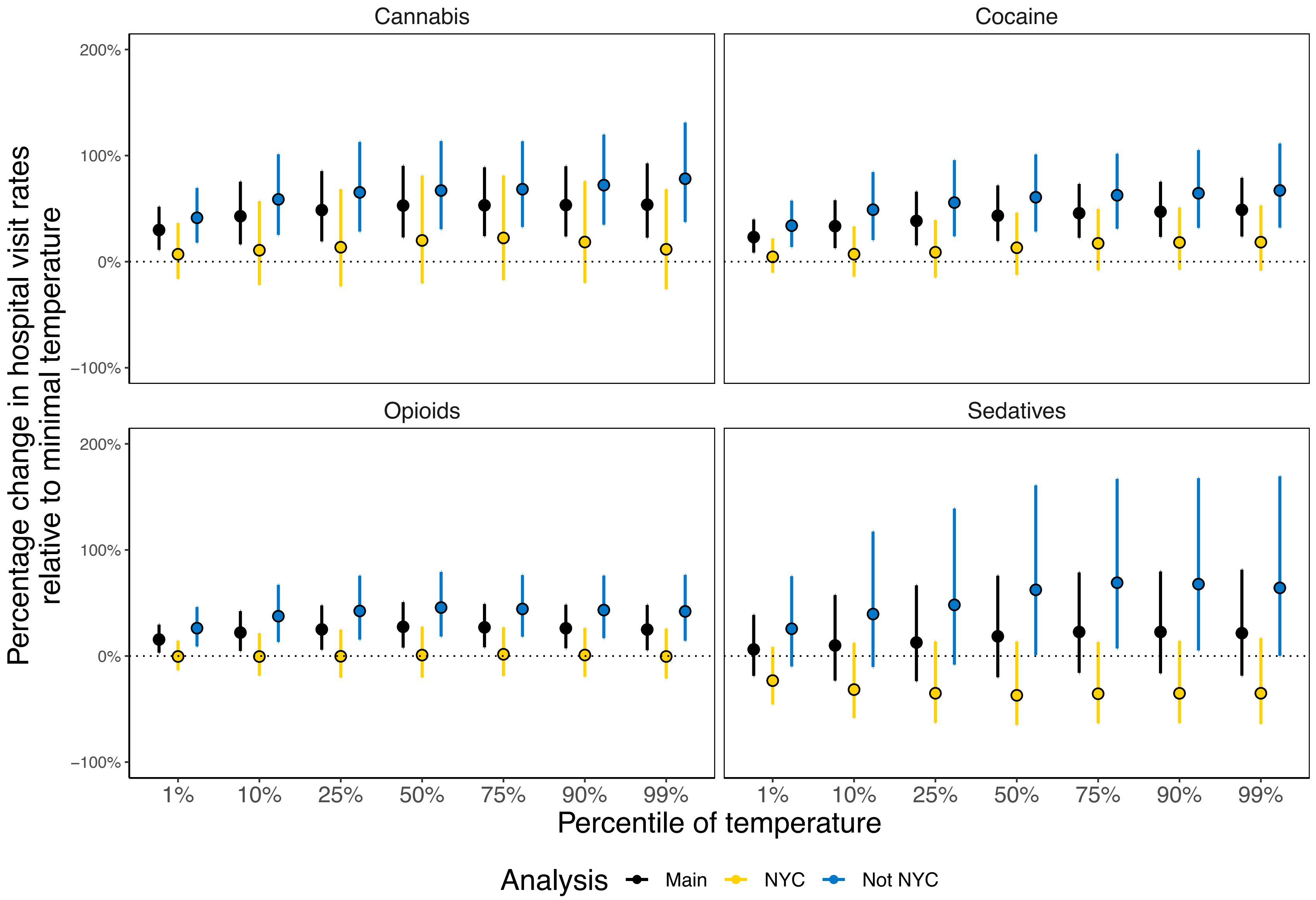

Supplementary Figure 3. Percentage change in hospital visit rates by selected percentiles of temperature relative to minimal temperature (-30.1°C (-22.18°F)) for cannabis, cocaine, opioid, and sedative hospital visits by location in New York State, were each of the lag days (0 to 6 days before) at the quoted temperature percentile before hospital visit. Points show the point estimates and whiskers represent 95% confidence intervals.

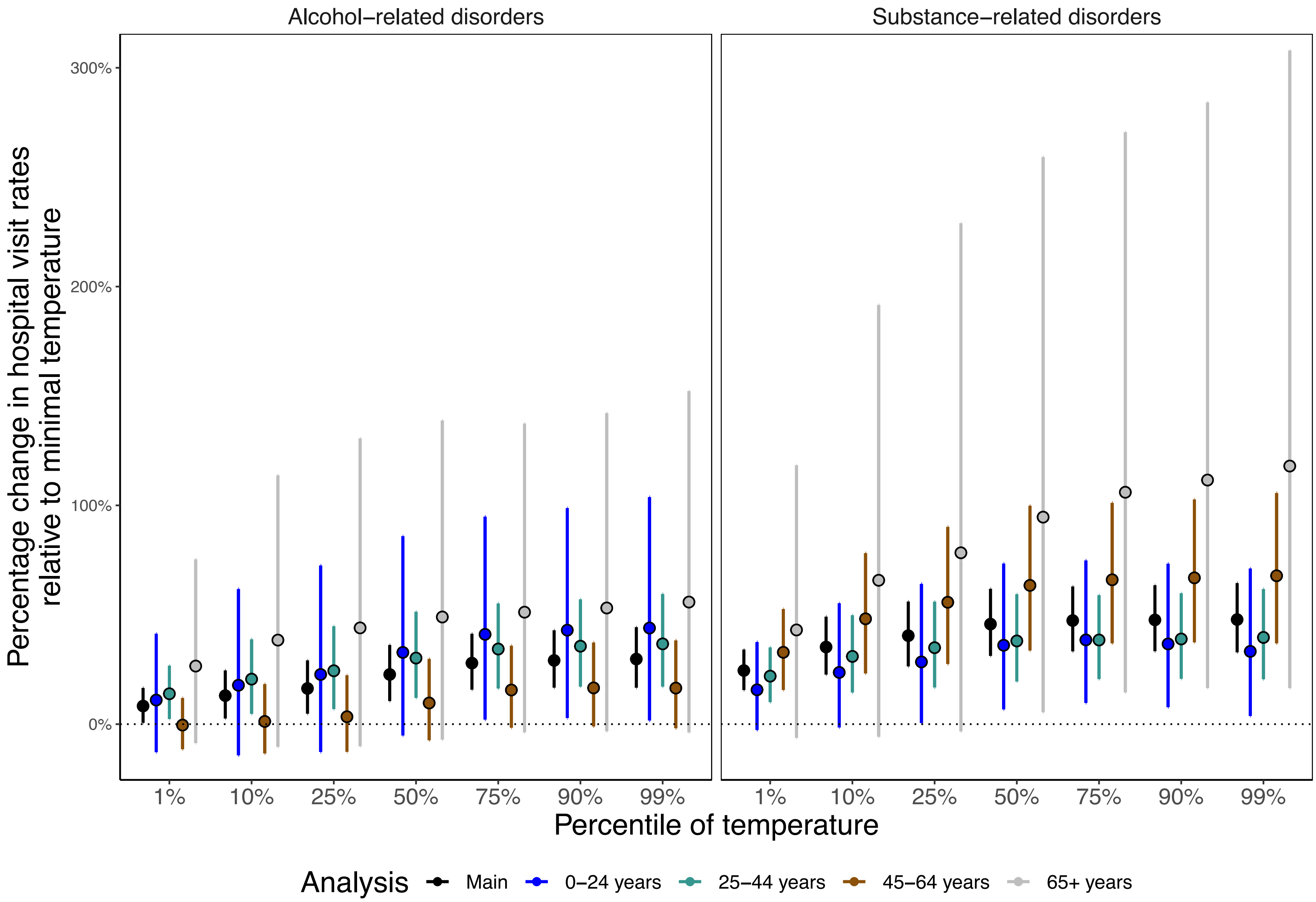

Supplementary Figure 4. Percentage change in hospital visit rates by selected percentiles of temperature relative to minimal temperature (-30.1°C (-22.18°F)) for alcohol- and substance-related disorder hospital visits by age group, were each of the lag days (0 to 6 days before) at the quoted temperature percentile before hospital visit. Points show the point estimates and whiskers represent 95% confidence intervals.

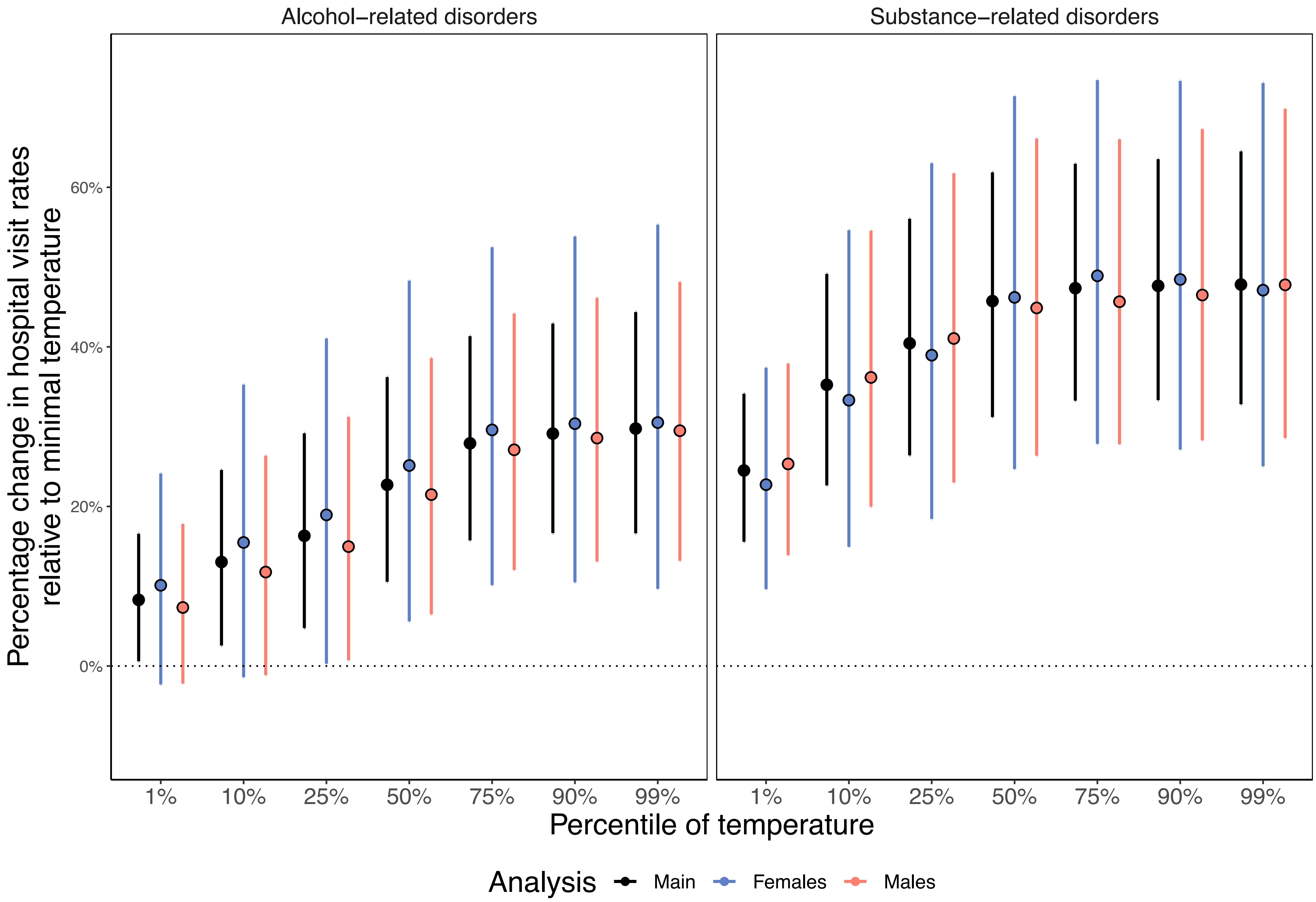

Supplementary Figure 5. Percentage change in hospital visit rates by selected percentiles of temperature relative to minimal temperature (-30.1°C (-22.18°F)) for alcohol- and substance-related disorder hospital visits by sex, were each of the lag days (0 to 6 days before) at the quoted temperature percentile before hospital visit. Points show the point estimates and whiskers represent 95% confidence intervals.

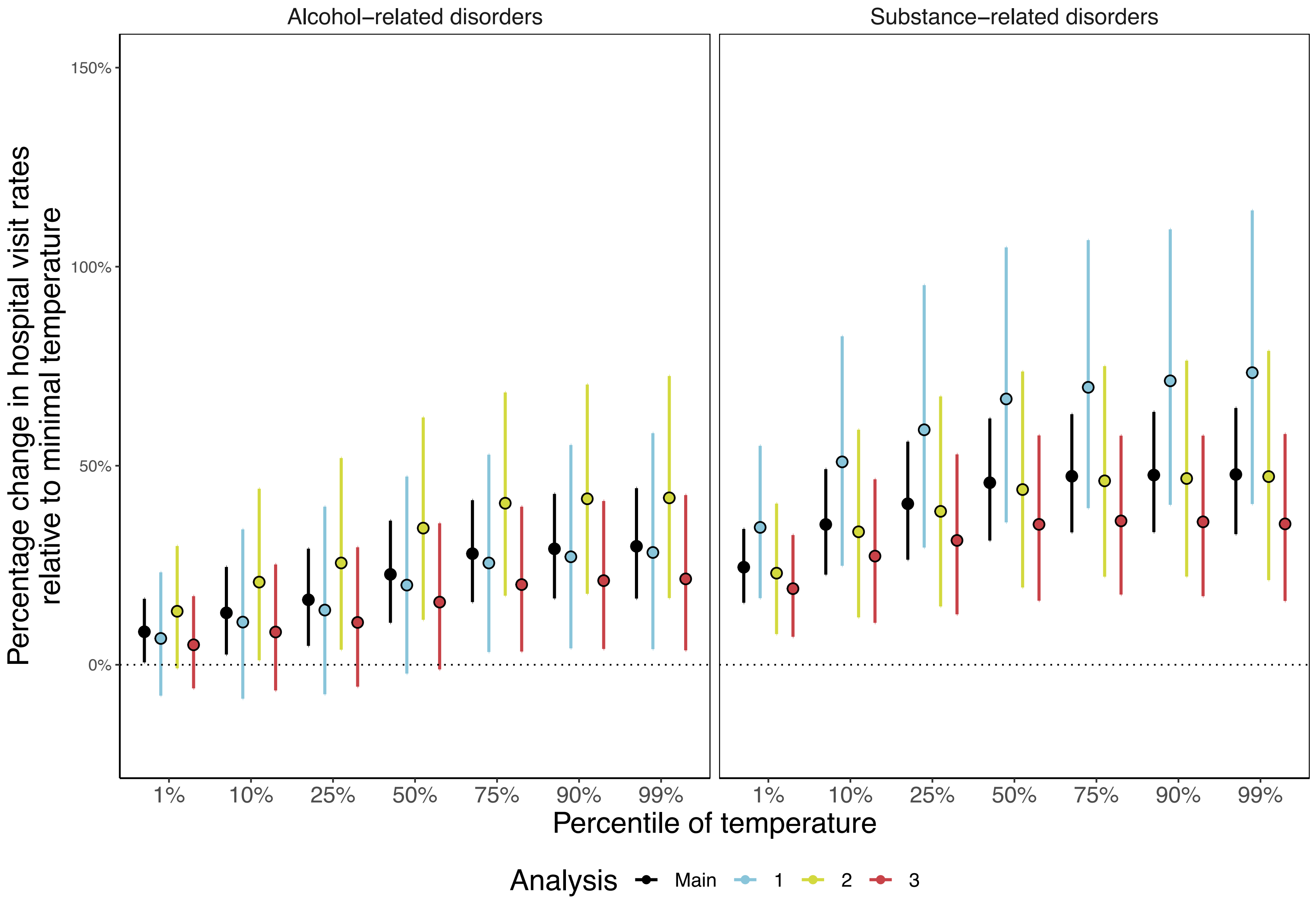

Supplementary Figure 6. Percentage change in hospital visit rates by selected percentiles of temperature relative to minimal temperature (-30.1°C (-22.18°F)) for alcohol- and substance-related disorder hospital visits by Social Vulnerability Index (SVI) tertile, were each of the lag days (0 to 6 days before) at the quoted temperature percentile before hospital visit. Points show the point estimates and whiskers represent 95% confidence intervals.

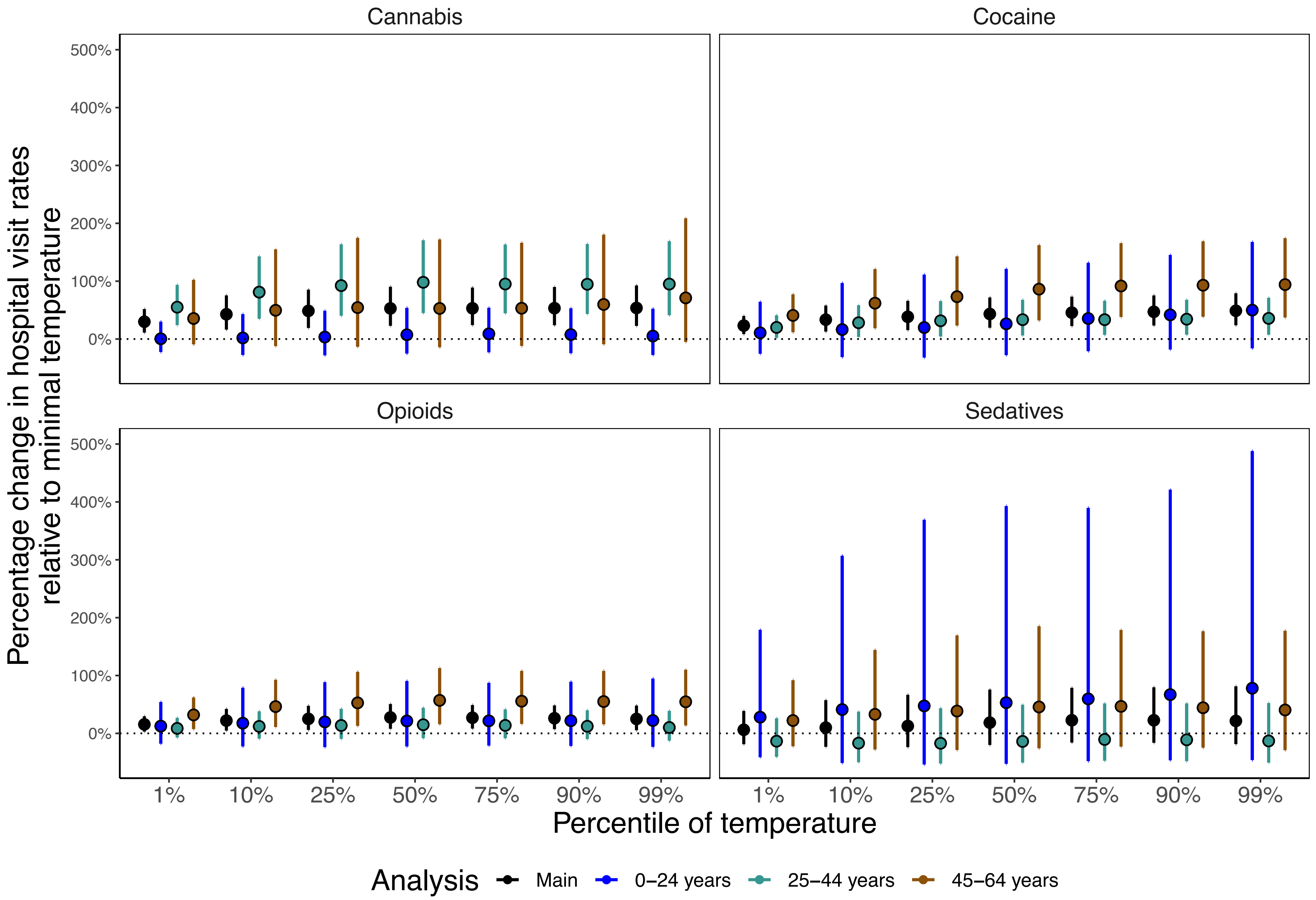

Supplementary Figure 7. Percentage change in hospital visit rates by selected percentiles of temperature relative to minimal temperature (-30.1°C (-22.18°F)) for cannabis, cocaine, opioid, and sedative hospital visits by age group, were each of the lag days (0 to 6 days before) at the quoted temperature percentile before hospital visit. Points show the point estimates and whiskers represent 95% confidence intervals.

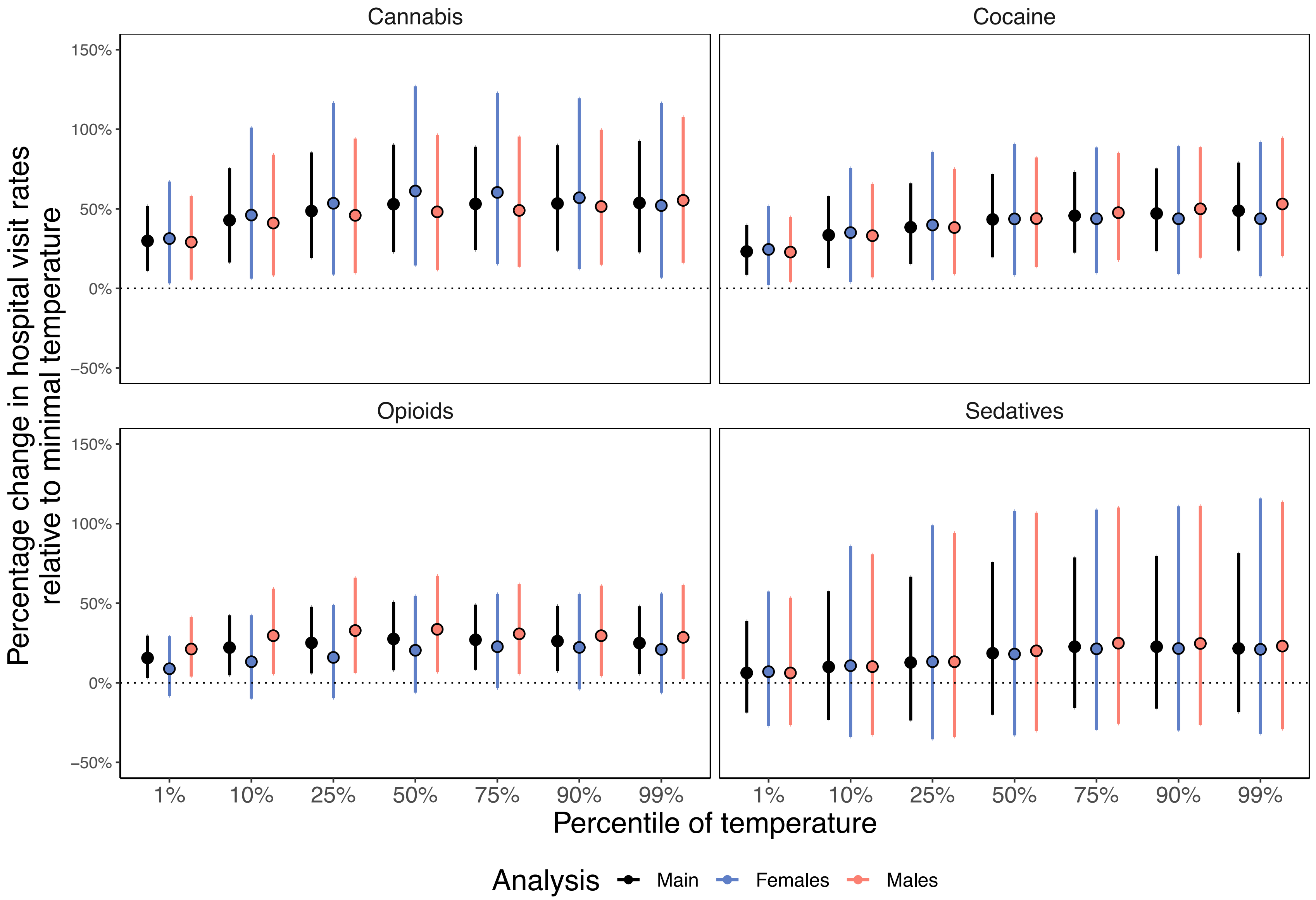

Supplementary Figure 8. Percentage change in hospital visit rates by selected percentiles of temperature relative to minimal temperature (-30.1°C (-22.18°F)) for cannabis, cocaine, opioid, and sedative hospital visits by sex, were each of the lag days (0 to 6 days before) at the quoted temperature percentile before hospital visit. Points show the point estimates and whiskers represent 95% confidence intervals.

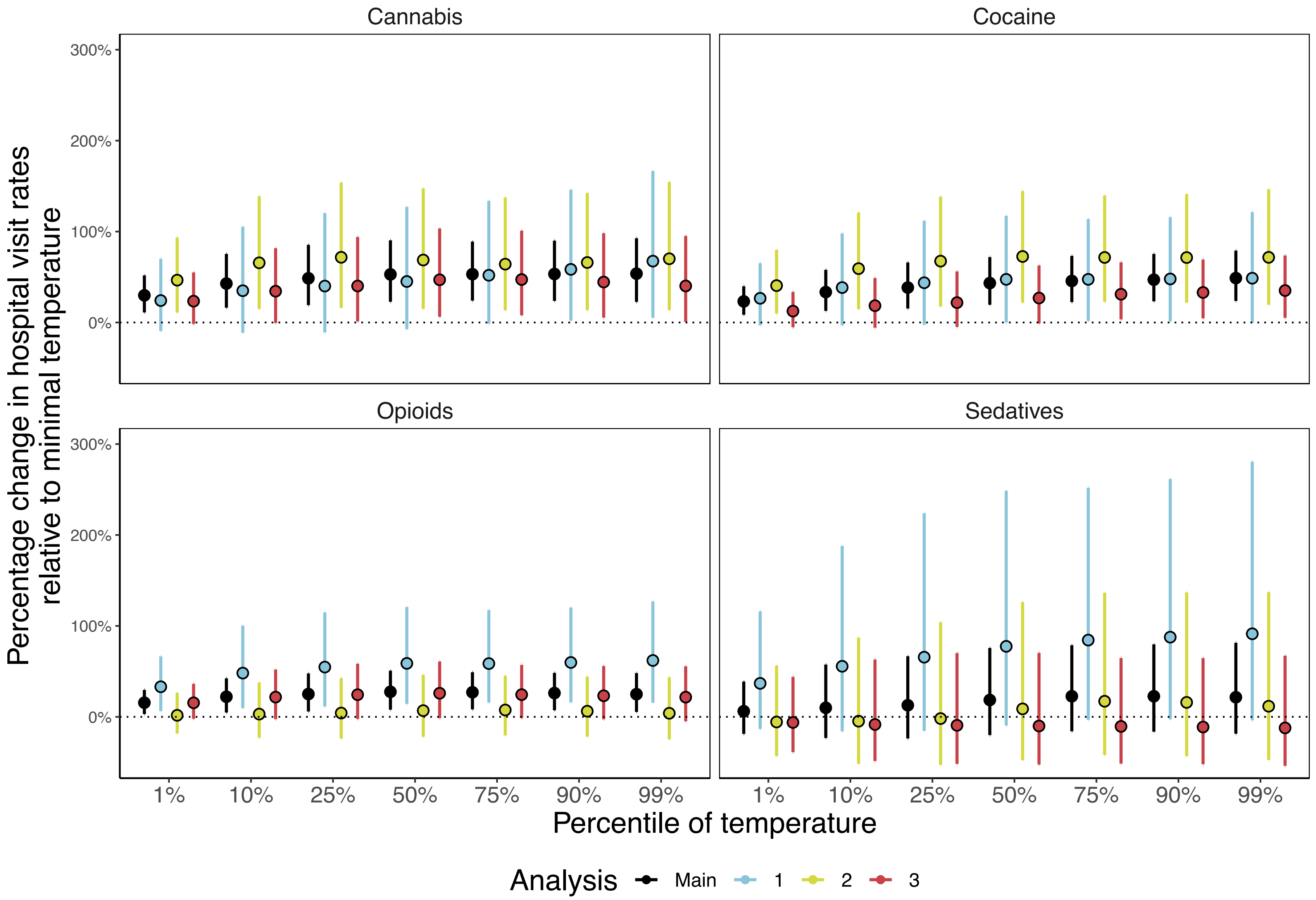

Supplementary Figure 9. Percentage change in hospital visit rates by selected percentiles of temperature relative to minimal temperature (-30.1°C (-22.18°F)) for cannabis, cocaine, opioid, and sedative hospital visits by Social Vulnerability Index (SVI) tertile, were each of the lag days (0 to 6 days before) at the quoted temperature percentile before hospital visit. Points show the point estimates and whiskers represent 95% confidence intervals.

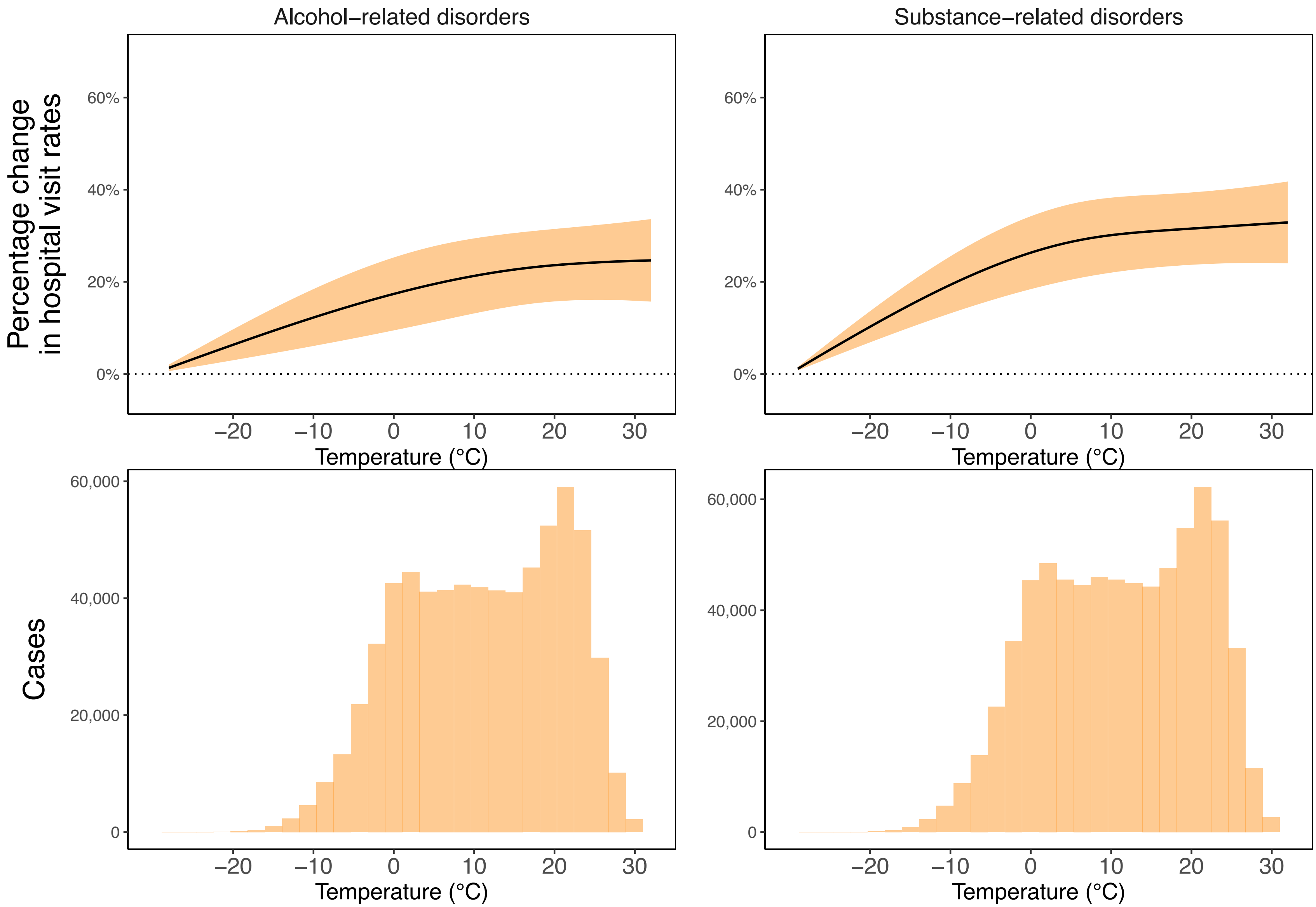

Supplementary Figure 10. Exposure-response curve of cumulative percentage change in hospital visit rates relative to minimal temperature (-30.1°C (-22.18°F)) for alcohol- and substance-related disorder hospital visits, were each of the lag days (0 to 1 day before) at the quoted temperature before hospital visit. Black lines show the point estimates and orange ribbons represent 95% confidence intervals.

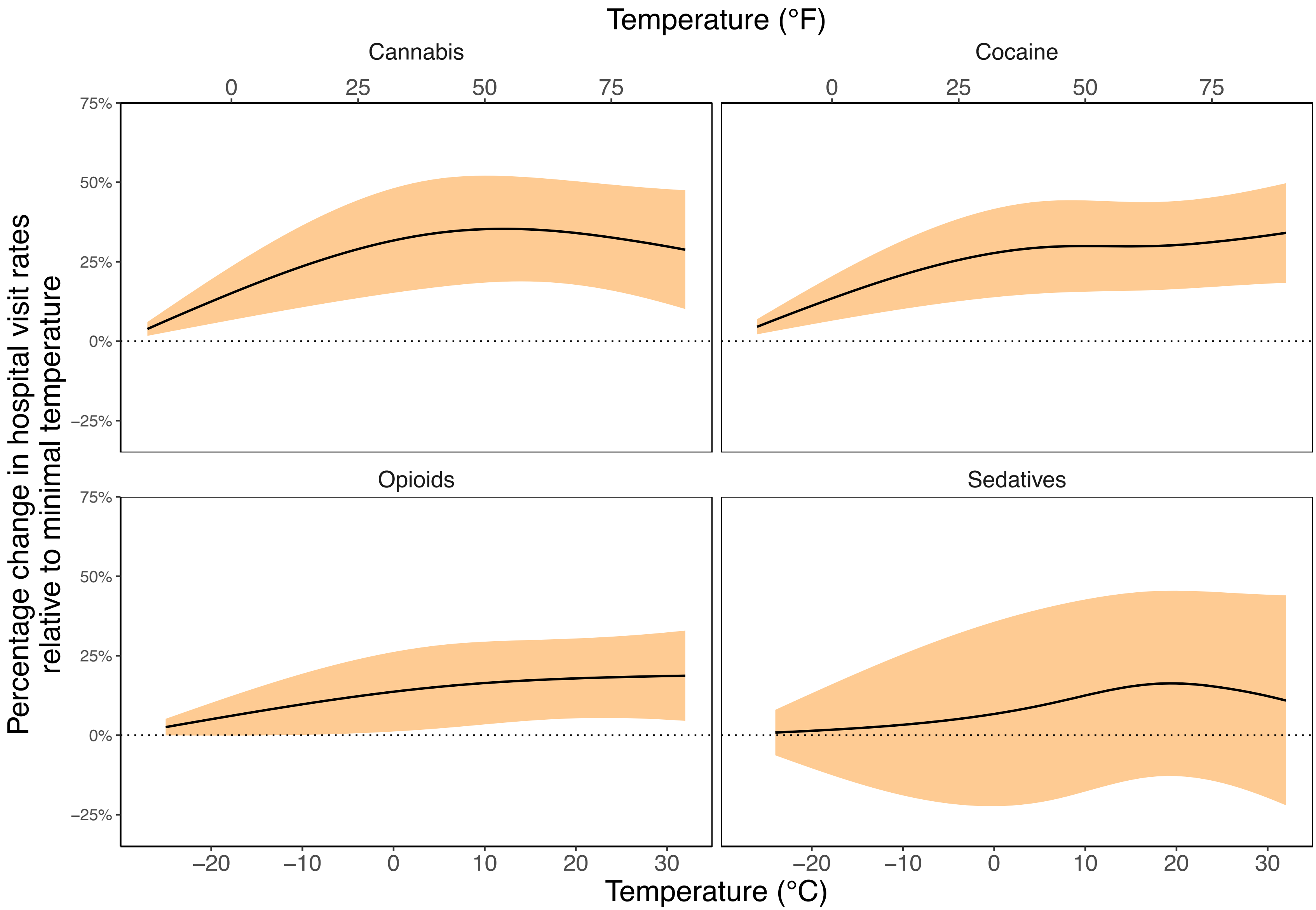

Supplementary Figure 11. Exposure-response curve of cumulative percentage change in hospital visit rates relative to minimal temperature (-30.1°C (-22.18°F)) for cannabis, cocaine, opioid, and sedative hospital visits, were each of the lag days (0 to 1 day before) at the quoted temperature before hospital visit. Black lines show the point estimates and orange ribbons represent 95% confidence intervals.

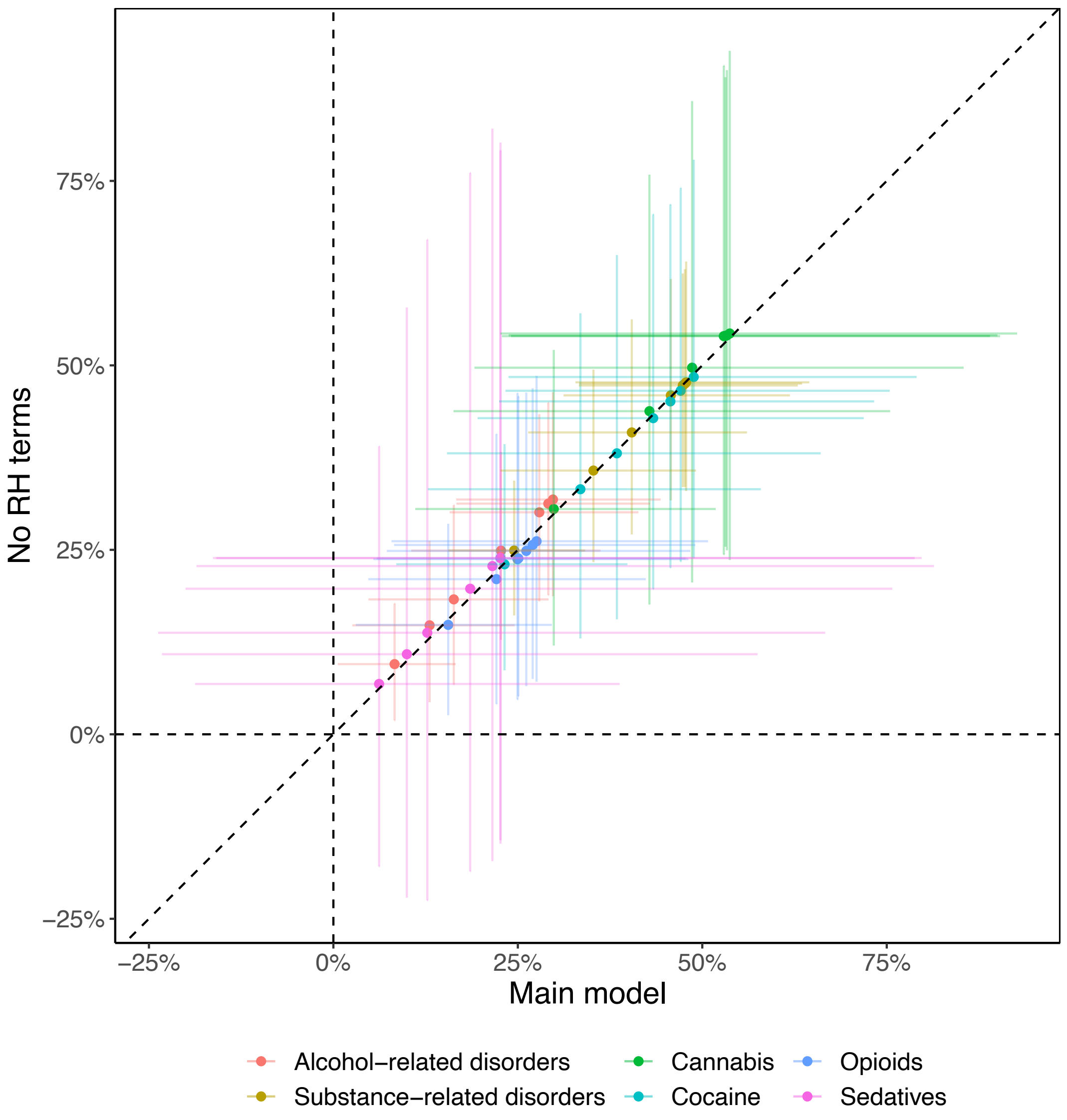

Supplementary Figure 12. Comparison of percentage change in hospital visit rates relative to minimal temperature (-30.1°C (-22.18°F)) by selected percentiles (1%, 10%, 25%, 50%, 75%, 90%, 99%) when including or not including relative humidity term ($\sum_{l=0}^{6} s(RH, df)_{lci}$), for a cumulative lag of up to six days before hospital visit. The values from the main model are on the x-axis with values from alternative model with no relative humidity terms on the y-axis. Dots show the point estimates and whiskers represent 95% credible intervals.

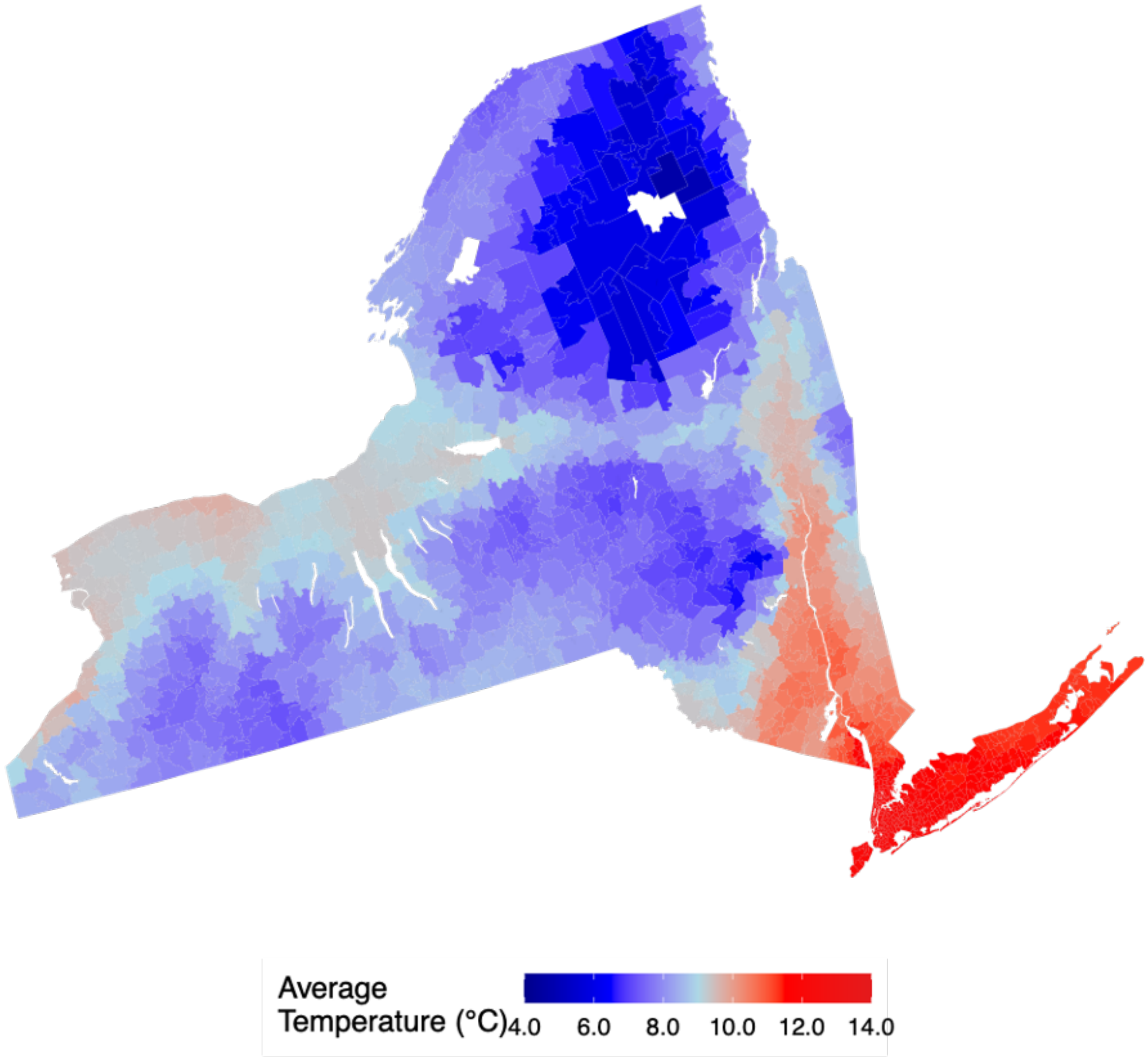

Supplementary Figure 13. Map of average temperature by ZIP Code in New York State for 1995-2014.

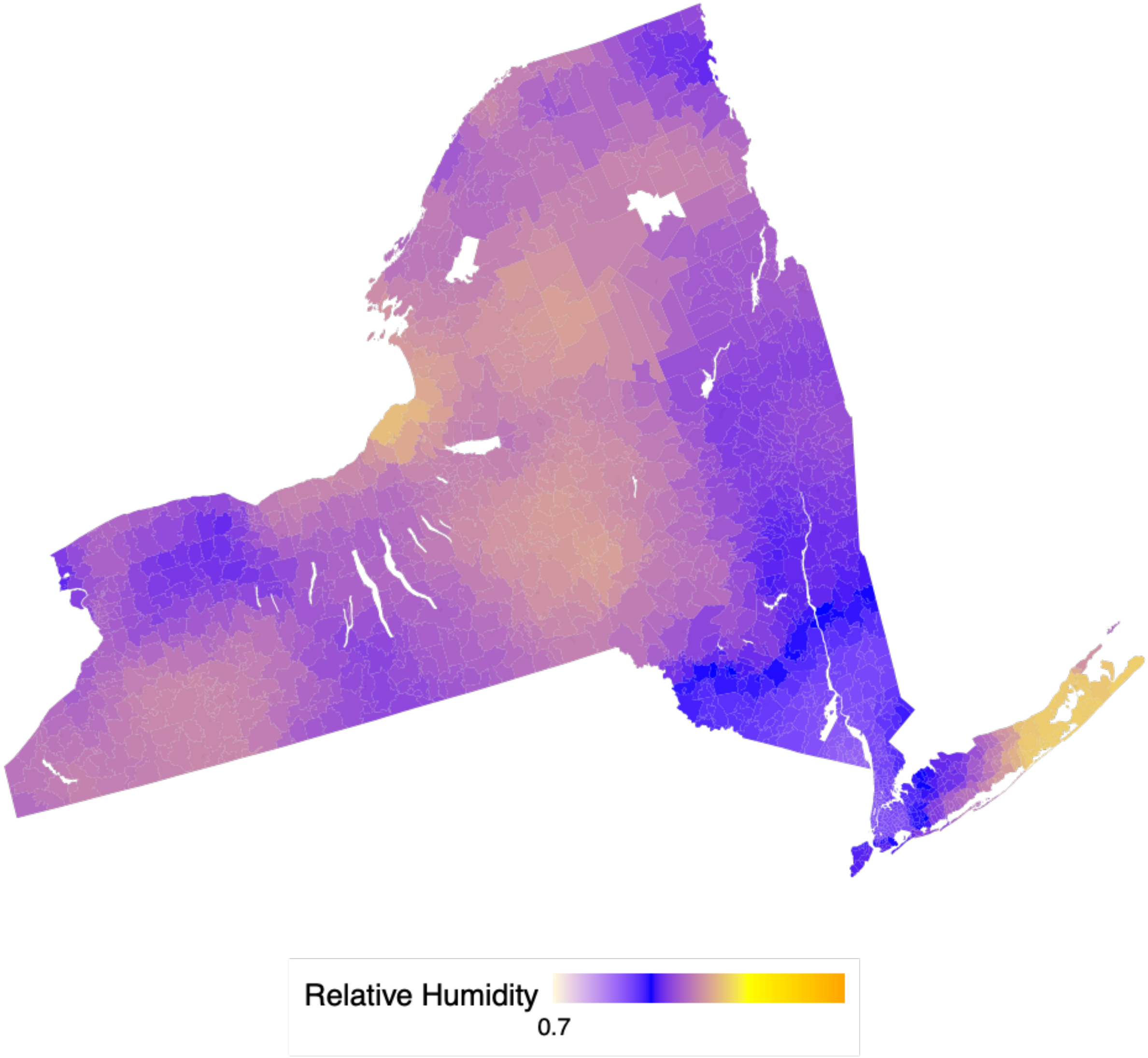

Supplementary Figure 14. Map of average relative humidity by ZIP Code in New York State for 1995-2014.

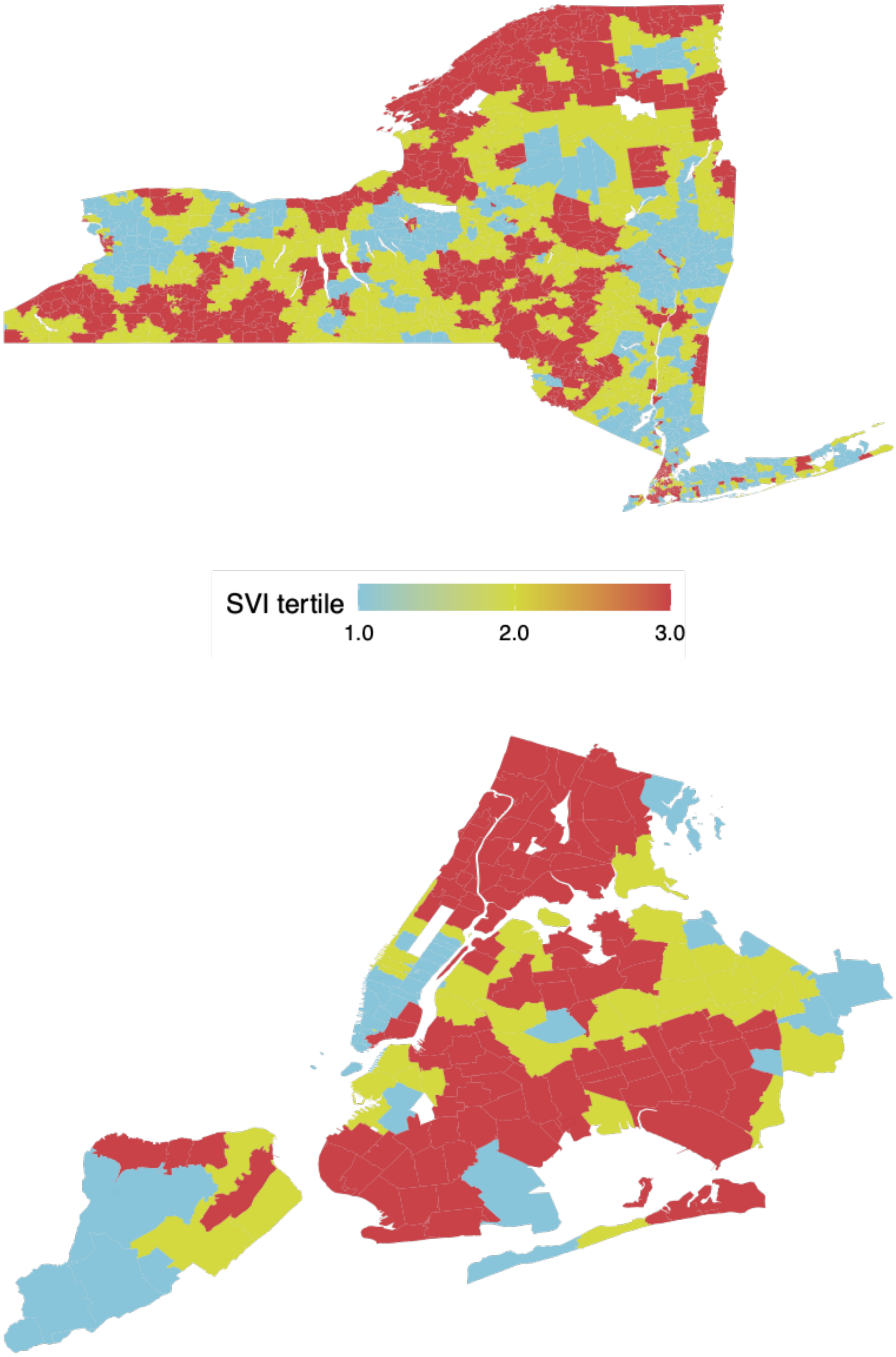

Supplementary Figure 15. 2014 Social Vulnerability Index (SVI) tertiles by ZIP Code in New York State. The first tertile (blue) represents lowest social vulnerability and the third tertile (red) represents highest social vulnerability.

Supplementary Table 1. Causes of hospital visit used in the analysis with ICD-9-CM codes.

Cause of hospital visit	ICD-9-CM
Alcohol-related disorders	291.0, 291.1, 291.2, 291.3, 291.4, 291.5, 291.8, 291.81, 291.82, 291.89, 291.9, 303.00, 303.01, 303.02, 303.03, 303.90, 303.91, 303.92, 303.93, 305.00, 305.01, 305.02, 305.03, 760.71, 980.0
Substance-related disorders	292.0, 292.11, 292.12, 292.2, 292.81, 292.82, 292.83, 292.84, 292.85, 292.89, 292.9, 304.00, 304.01, 304.02, 304.03, 304.10, 304.11, 304.12, 304.13, 304.20, 304.21, 304.22, 304.23, 304.30, 304.31, 304.32, 304.33, 304.40, 304.41, 304.42, 304.43, 304.50, 304.51, 304.52, 304.53, 304.60, 304.61, 304.62, 304.63, 304.70, 304.71, 304.72, 304.73, 304.80, 304.81, 304.82, 304.83, 304.90, 304.91, 304.92, 304.93, 305.20, 305.21, 305.22, 305.23, 305.30, 305.31, 305.32, 305.33, 305.40, 305.41, 305.42, 305.43, 305.50, 305.51, 305.52, 305.53, 305.60, 305.61, 305.62, 305.63, 305.70, 305.71, 305.72, 305.73, 305.80, 305.81, 305.82, 305.83, 305.90, 305.91, 305.92, 305.93, 648.30, 648.31, 648.32, 648.33, 648.34, 655.50, 655.51, 655.53, 760.72, 760.73, 760.75, 779.5, 965.00, 965.01, 965.02, 965.09, V65.42
Cannabis	304.30, 304.31, 304.32, 304.33, 305.20, 305.21, 305.22, 305.23
Cocaine	304.20, 304.21, 304.22, 304.23, 304.40, 304.41, 304.42, 304.43, 305.60, 305.61, 305.62, 305.63, 305.70, 305.71, 305.72, 305.73, 305.80, 305.81, 305.82, 305.83
Opioids	304.00, 304.01, 304.02, 304.03, 304.70, 304.71, 304.72, 304.73, 305.50, 305.51, 305.52, 305.53, 965.00, 965.01, 965.02, 965.09
Sedatives	304.10, 304.11, 304.12, 304.13, 305.40, 305.41, 305.42, 305.43
