# Supplementary material for: The association between temperature and alcohol- and substance-related disorder hospital visits in New York State
Source: Commun Med (Lond). 2023 Sep 26;3:118. doi: 10.1038/s43856-023-00346-1 (PMC10522658; doi:10.1038/s43856-023-00346-1)
Supplement: Supplementary file 3 — Reporting Summary [file 43856_2023_346_MOESM3_ESM.pdf]

nature portfolio

Corresponding author(s): Robbie M. Parks

Last updated by author(s): Jul 12, 2023

Reporting Summary

Nature Portfolio wishes to improve the reproducibility of the work that we publish. This form provides structure for consistency and transparency in reporting. For further information on Nature Portfolio policies, see our Editorial Policies and the Editorial Policy Checklist.

Statistics

For all statistical analyses, confirm that the following items are present in the figure legend, table legend, main text, or Methods section.

n/a	Confirmed	
☐	☒	The exact sample size (*n*) for each experimental group/condition, given as a discrete number and unit of measurement
☐	☒	A statement on whether measurements were taken from distinct samples or whether the same sample was measured repeatedly
☐	☒	The statistical test(s) used AND whether they are one- or two-sided<br>*Only common tests should be described solely by name; describe more complex techniques in the Methods section.*
☐	☒	A description of all covariates tested
☐	☒	A description of any assumptions or corrections, such as tests of normality and adjustment for multiple comparisons
☐	☒	A full description of the statistical parameters including central tendency (e.g. means) or other basic estimates (e.g. regression coefficient) AND variation (e.g. standard deviation) or associated estimates of uncertainty (e.g. confidence intervals)
☐	☒	For null hypothesis testing, the test statistic (e.g. *F*, *t*, *r*) with confidence intervals, effect sizes, degrees of freedom and *P* value noted<br>*Give P values as exact values whenever suitable.*
☐	☒	For Bayesian analysis, information on the choice of priors and Markov chain Monte Carlo settings
☐	☒	For hierarchical and complex designs, identification of the appropriate level for tests and full reporting of outcomes
☐	☒	Estimates of effect sizes (e.g. Cohen's *d*, Pearson's *r*), indicating how they were calculated

Our web collection on statistics for biologists contains articles on many of the points above.

Software and code

Policy information about availability of computer code

Data collection	Hospital records were obtained across NYS from 1995 to 2014 from the New York Department of Health Statewide Planning and Research Cooperative System (SPARCS) (https://www.health.ny.gov/statistics/sparcs/).<br><br>Daily average temperature, specific humidity, and pressure were obtained from the North American Land Data Assimilation System, NLDAS-2 Forcing,28 with full space and time coverage over the study period.
Data analysis	Statistical analyses were conducted using the R Statistical Software, version 4.1.1,38 and dlnm, version 2.4.2.

For manuscripts utilizing custom algorithms or software that are central to the research but not yet described in published literature, software must be made available to editors and reviewers. We strongly encourage code deposition in a community repository (e.g. GitHub). See the Nature Portfolio guidelines for submitting code & software for further information.

Data

Policy information about availability of data

All manuscripts must include a data availability statement. This statement should provide the following information, where applicable:

- Accession codes, unique identifiers, or web links for publicly available datasets
- A description of any restrictions on data availability
- For clinical datasets or third party data, please ensure that the statement adheres to our policy

NLDAS-2 temperature, specific humidity, and pressure data are downloadable from https://disc.sci.gsfc.nasa.gov/datasets?keywords=NLDAS. SPARCS hospital records can be requested through submission of a proposal to the New York State Department of Health (https://www.health.ny.gov/statistics/sparcs/).

All code for analysis, results from analysis, and visualization presented in this manuscript will be publicly available via GitHub.

Research involving human participants, their data, or biological material

Policy information about studies with human participants or human data. See also policy information about sex, gender (identity/presentation), and sexual orientation and race, ethnicity and racism.

Reporting on sex and gender	In addition to the main analyses investigating all hospital visits together for each cause and sub-cause (alcohol-related disorders, substance-related disorders, cannabis, cocaine, opioids and sedatives), further assessment was made of whether estimated effects varied by location (NYC or not NYC), sex (female or male), age group (0-24 years, 25-44 years, 45-64 years or 65+ years), or by SVI tertile (low vulnerability to high vulnerability, 1 to 3), by conducting stratified analyses, using the same model as described above.
Reporting on race, ethnicity, or other socially relevant groupings	In addition to the main analyses investigating all hospital visits together for each cause and sub-cause (alcohol-related disorders, substance-related disorders, cannabis, cocaine, opioids and sedatives), further assessment was made of whether estimated effects varied by location (NYC or not NYC), sex (female or male), age group (0-24 years, 25-44 years, 45-64 years or 65+ years), or by SVI tertile (low vulnerability to high vulnerability, 1 to 3), by conducting stratified analyses, using the same model as described above.
Population characteristics	For each admission record, International Classification of Diseases, Ninth Revision, Clinical Modification (ICD-9-CM) diagnosis codes were obtained, along with patient residential ZIP Code, date of admission, age, and sex.
Recruitment	Observational study so no recruitment
Ethics oversight	This study was approved by the Institutional Review Board at the Columbia Mailman School of Public Health and was classified as exempt from needing to obtain Informed Consent (Protocol IRB-AAAR0877).

Note that full information on the approval of the study protocol must also be provided in the manuscript.

Field-specific reporting

Please select the one below that is the best fit for your research. If you are not sure, read the appropriate sections before making your selection.

☒ Life sciences ☐ Behavioural & social sciences ☐ Ecological, evolutionary & environmental sciences

For a reference copy of the document with all sections, see nature.com/documents/nr-reporting-summary-flat.pdf

Life sciences study design

All studies must disclose on these points even when the disclosure is negative.

Sample size	Full sample of all hospital visits in NYS from 1995-2014.
Data exclusions	Admissions with missing, incomplete, or inaccurate records of sex, age, dates of admission, or residential ZIP Codes were excluded (16.4% of alcohol-related disorder and 9.2% of substance-related disorder hospital visits).
Replication	N/A
Randomization	N/A
Blinding	N/A

Reporting for specific materials, systems and methods

We require information from authors about some types of materials, experimental systems and methods used in many studies. Here, indicate whether each material, system or method listed is relevant to your study. If you are not sure if a list item applies to your research, read the appropriate section before selecting a response.

Materials & experimental systems

n/a	Involved in the study
☒	☐ Antibodies
☒	☐ Eukaryotic cell lines
☒	☐ Palaeontology and archaeology
☒	☐ Animals and other organisms
☒	☐ Clinical data
☒	☐ Dual use research of concern
☒	☐ Plants

Methods

n/a	Involved in the study
☒	☐ ChIP-seq
☒	☐ Flow cytometry
☒	☐ MRI-based neuroimaging
